# One-stage sex reassignment surgery at the delayed presentation in a patient with partial androgen insensitivity syndrome: A case report

**DOI:** 10.1016/j.ijscr.2021.106355

**Published:** 2021-08-26

**Authors:** Pham Thi Viet Dung, Tran Thiet Son, Phan Van Tan

**Affiliations:** a Department of Plastic Surgery, Hanoi Medical University, No.1 Ton That Tung Street, Hanoi, Viet Nam; b Department of Platic Reconstructive and Aesthetic Surgery, Hanoi Medical University Hospital, No.1 Ton That Tung Street, Hanoi, Viet Nam; c Department of Platic Reconstructive and Aesthetic Surgery, Bach Mai hospital, No.78 Giai Phong Street, Hanoi, Viet nam

**Keywords:** Partial androgen insensitivity syndrome, Gonadectomy, Clitoroplasty, Vaginoplasty, Sex reassignment

## Abstract

**Introduction and importance:**

The partial androgen insensitivity syndrome (PAIS) is a rare genetic disorder, which needs to be diagnosed early and provided suitable treatment. One-stage sex reassignment surgery can be considered as one of the treatment options for PAIS patients.

**Case presentation:**

A 44-year-old patient with PAIS was admitted to our hospital. After getting a consultation, the patient decided to choose the one-stage sex reassignment surgery to be reassigned to be a female. The surgery consisted of breast augmentation and genital surgery. After 8 months of follow-up, the patient's breast had a desired shape and volume. The clitoris was in normal size with normal sensation, and the neovagina was 8 cm in depth with a smooth mucosal surface. We also observed that the minor labia were symmetric. The patient reported achieving orgasms with sex toys.

**Clinical discussion:**

The one-stage sex reassignment surgery for the PAIS patient is safe and reduces treatment time for patients. It could also bring many benefits to the patients, such as reducing the incision, preventing gonadoblastoma and giving a sense of the patient's female gender which helps the patient feel confident and improve her quality of life. Thus, the one-stage surgery should be indicated for the patient at middle-aged who shouldn't be delayed anymore to have normal female breast and external genitalia.

**Conclusion:**

The one-stage sex reassignment surgery was performed safely and successfully on the delayed presentation of the PAIS patient. This could be an effective and appropriate approach to treat late-diagnosed PAIS patients.

## Highlights

• One-stage sex reassignment surgery for a patient with partial androgen insensitivity syndrome
• A 44-year-old patient at the delayed presentation of the disorder
• We observed good treatment outcomes with a 8-moth follow-up

## 1 Introduction

The partial androgen insensitivity syndrome (PAIS) is a rare genetic disorder, which is one phenotype of androgen insensitivity syndrome (AIS). This disorder leads to the partial inability of cell response to androgen. Patients with PAIS have phenotype 46, XY, but they appear with ambiguous genitalia at birth, primary amenorrhea with clitoromegaly, inguinal masses, without Mullerian structures [1], [2], [3], [4]. After the patients have puberty, the treatment for this disorder depends on the expected sex of the patients, in which they usually desire the gender that belongs to the dominant external genitalia phenotype and the assigned gender at birth [5]. Treatment for patients with PAIS needs the cooperation of multidiscipline specialists in endocrinology, gynecology, psychology and plastic surgery. The reconstruction of the breast and external genitalia plays an important role in eliminating body defects [5]. The PAIS patients can be assigned or reassigned sex by surgical intervention, and the appropriate time to do this is right after the patient's puberty. The PAIS patients with delayed diagnosis may have fewer chances of external genitalia reconstruction [6]. There are several possible approaches for the PAIS patients, of which one-stage sex reassignment surgery should be chosen to reduce treatment time [7]. This article presents a one-stage surgical sex reassignment at the delayed presentation in a PAIS patient. This report has been written in accordance with SCARE guidelines criteria [8].

## 2 Case presentation

A 44-year-old patient with female gender on the birth certificate was admitted to our hospital complaining of abnormal genitalia. The patient was found to have clitoromegaly from birth; however, she experienced amenorrhea after puberty. As an adult, the patient felt that she had not developed normally, so she attempted suicide at 20 years old. The patient identified herself as female, and she reported that she had a boyfriend that lasted for 5 years, but she broke up that relationship. Until admitted to our hospital, the patient had never been examined and treated. The patient had a brother and a sister; they both had normal external genitalia and had children.

**Fig. 1:**
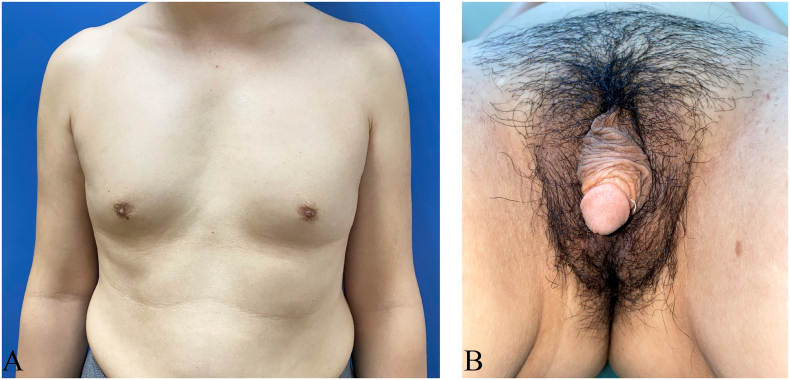
Shows the appearance of the patients' patient's chest and external genitalia. (A) The breast developed in Tanner grade III. (B) External genitalia.

Fig. 1 shows the appearance of the patient's chest and external genitalia. In the flaccid stage, the penis defined as a clitoris was 4.5 cm in length and 2 cm in diameter. The scrotums looked like labia majora, and the urethra meatus was located at the base of the penis. Masses located at the inguinal canal on both sides were determined testis. The patients had blind-ending vagina with 1 cm in length and 5 mm in width. The patient had undermasculinization phenotype with pubic hair in Tanner grade III, and her breasts developed at Tanner grade III.

**Fig. 2:**
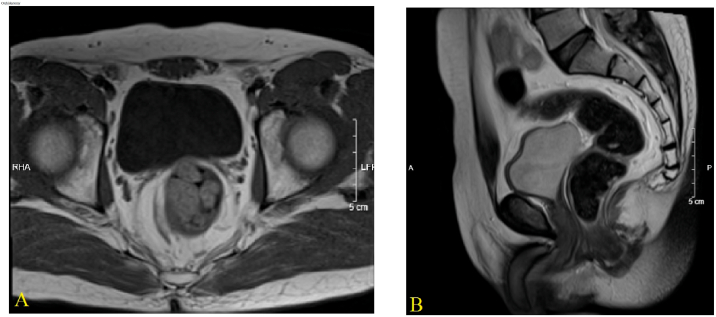
MRI shows no ovary, fallopian tube, uterus and superior part of the vagina, prostate and seminal vesicles. (A) MRI with cross section. (B) MRI with longitudinal section.

Fig. 1 shows the appearance of the patient's chest and external genitalia with masculinization phenotype. In the flaccid stage, the penis defined as a clitoris was 4.5 cm in length and 2 cm in diameter. The scrotums looked like labia majora, and the urethra meatus was located at the base of the penis. Masses located at the inguinal region on both sides, which were determined as testis. Her breasts and public hair developed at Tanner grade III. Magnetic resonance imaging (MRI) revealed the absence of internal female genitalia, such as ovaries, fallopian tubes, uterus, superior part of the vagina (Fig. 2). Serum testosterone levels were 2.49 nmol/L, showing higher than that of the normal range of 0.29–1.67 nmol/L. Other serum sex hormone levels were in the normal range. In addition, the blood karyotyping showed 46, XY pattern. The patient was diagnosed to be a case of PAIS.

**Fig. 3:**
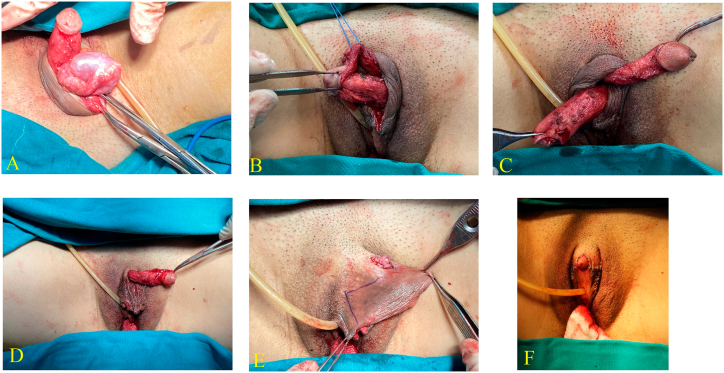
Shows the performance of orchidectomy, penectomy, clitoris and minor labia reconstruction. (A) Orchidectomy. (B) and (C) Corpus cavernosa and spongiosum were resected by Kogan technique. (D) The glans was passed through a slit on penis skin. (E) The skin was severed to reconstruct labia minora (F) Externalgenitaliaa after the operation.

**Fig. 4:**
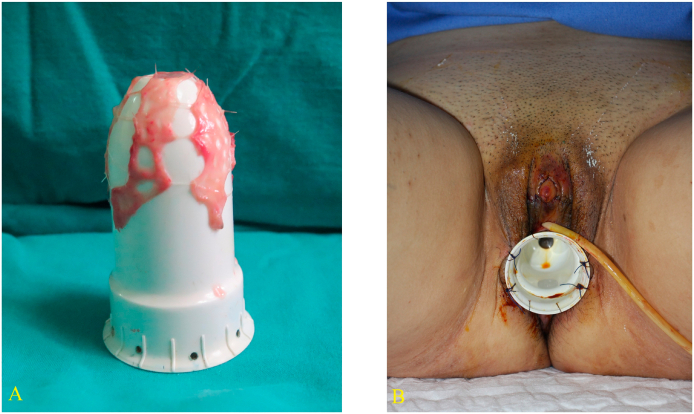
Shows the performance of vaginoplasty by autologous buccal mucosal graft. (A) Meshing autologous buccal mucosal was put on a dilator. (B) Immobilizing dilator to neovagina.

After receiving the consultation, the patient decided to reassign a female because she had an attraction toward male characters. The procedure was carried out by a senior surgeon with more than 16 years of surgical experience in the field. A one-stage surgical sex reassignment was performed with breast augmentation and external genital surgery. The breast augmentation was performed with two micro-texture implants with medium projection, in which each implant had 310 mL in volume, and was placed at the subpectoral plane through periareolar incisions. Fig. 3 presents the performance of gonadectomy, penectomy, clitoris and minor labia reconstruction. The orchidectomy was conducted by applying a circumference foreskin incision. The penis's skin was degloved and testes were exposed at external inguinal rings, showing that the testes had normal shape, hypoplasia, dilated epididymis. The penectomy was done with resecting corpus cavernosa and spongiosum by applying Kogan's technique [9]. In the clitoroplasty and minor labial reconstruction, the glans was reduced to the size to correspond to the clitoris, and passed through a slit 1 cm on penis skin at the penis base, and then it was sutured to the borders of the slit to complete the clitoroplasty. Fig. 4 shows the performance of vaginoplasty by using mesh autologous buccal mucosal graft and immobilizing dilator to neovagina. The vaginal cavity was dissected with 8 cm in depth and 4 cm in diameter. Two pieces of buccal mucosa were harvested from in superior lip and cheek with the size of 1.5 cm × 14 cm, and the inferior lip with the size of 1.5 cm × 12 cm. Several pinhole-sized incisions were made to enlarge buccal mucous grafts. Then grafts were stretched and wrapped around the dilator. The dilator was inserted into the vaginal cavity. Finally, the dilator was sutured to immobilize the graft and ensure it remained in place.

**Fig. 5:**
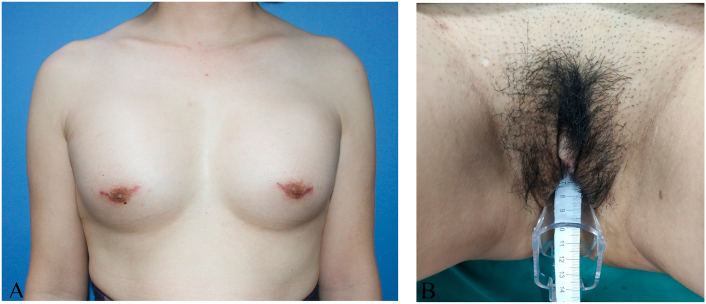
Shows follow-up results. (A) Breast augmentation after 1.5 months. (B) Vagina depth was 8 cm when examining by speculum after 8 months.

The total time for one-stage surgical sex reassignment was approximately 4.5 h. The urinary catheter and dilator were removed on postoperative day 10. The results of testicular biopsy showed fibrosis seminiferous tubules without malignant cells. The patient was discharged at 14 days postoperatively and was advised to have sexual intercourse after 6 weeks. After 8 months of follow-up, the patient reported not having any sexual partner; however, she still frequently dilated the vaginal cavity with a sex toy. The clitoris maintained appropriate size and sensitivity without numbness, pain, or erection. Fig. 5 shows the breast augmentation after 1.5 months and vaginal depth of 8 cm after 8 months. The neovagina was 8 cm in-depth, with a smooth mucosal surface when examined by a speculum dilator. Labia majora and labia minora are symmetric. The patient reported that she achieved orgasm sensations when using a sex toy. Sex hormone levels in serum were within the normal range at 8 months after surgery.

## 3 Discussion

This is the first case treated by a one-stage sex reassignment surgery at the delayed presentation in a PAIS patient to be reported in Vietnam. We have found that this is a safe approach and reduces treatment time for patients. In particular, the patient got the desired sex, through which the patient felt more confident and improved her quality of life. We think this will be a lesson in applying one-stage sex reassignment surgery to similar cases of PAIS patients.

The patient was not diagnosed and treated early due to difficult economic circumstances and fear of social stereotypes. In addition, the patient had to give up relationships due to feelings of self-deprecation and guilt complex. The patient also felt stuck and tried to commit suicide, but failed. After consulting with us and recommending one-stage sex reassignment surgery, it took the patient months to decide. The main motivation for the patient to make the decision to undergo surgery is to want to change her life, overcome societal prejudices and find new opportunities in her relationship.

We performed the one-stage sex reassignment surgery with many procedures performed, but it brought several benefits to the patient. In this case, the combination of orchidectomy with penectomy allowed us to reduce an incision by resecting testes after degloving the penis skin and exposing the testes [5]. The incidence of gonadoblastoma in PAIS patients has been reported to range from 9.5 to 16.7%, indicating that orchidectomy is essential for the patient [10], [11]. Thus, the orchidectomy has often been recommended to be performed early, preferably shortly after puberty. If the orchiectomy cannot be performed, the patient can also solve this problem by doing a biopsy to monitor [12], [13], but this will bring disadvantages. In addition, the indication of orchidectomy in PAIS can be considered if testes located in the inguinal area, which cause discomfort to the patient's life. In our case, the patient was monitored and managed by an endocrinologist after undergoing orchiectomy. The patient's serum sex hormone levels were within the normal range, so hormone treatment therapy was not recommended by this time.

Vaginoplasty surgery is one of the important procedures to give a sense of the patient's female gender. The basic principle of vaginoplasty surgery is to create a vaginal cavity and to cover the inner side to prevent it from healing. There have been many methods applied to solve the problem of covering the surface of the vaginal cavity [4], [5]; however, we chose vaginoplasty surgery by using mesh autologous buccal mucosal graft due to its many advantages. We created slits in mucosa pieces to enlarge it as mesh thin skin graft. Our team has successfully implemented this technique and was published in a journal in Vietnam in 2016 and 2020 [14], [15]. In previous studies using the same material for diagnosed vaginal agenesis, the authors also reported that this was a simple and effective method for vaginoplasty surgery [16], [17].

## 4 Conclusion

In the present case, the one-stage sex reassignment surgery was performed safely and successfully on the delayed presentation of the PAIS patient. More studies may be needed to compare and determine the role of one-stage sex reassignment surgery in the delayed presentation of the PAIS patient. However, we believe that this is a suitable method to save treatment time, especially since it has satisfied the patient.

## Abbreviation

AIS: Androgen insensitivity syndrome
PAIS: Partial androgen insensitivity syndrome

## Sources of funding

None.

## Ethical approval

Approval is not necessary for a case report in our locality.

## Consent

Written informed consent was obtained from the patient for publication of this case report and accompanying images. A copy of the written consent is available for review by the Editor-in-Chief of this journal on request.

## Author contribution

Pham Thi Viet Dung: first and corresponding author, performed the operation, conceptualization, writing and revising the manuscript.

Phan Van Tan: writing and revising the manuscript.

Tran Thiet Son: reviewing and editing the manuscript.

## Registration of research studies

N/A.

## Guarantor

Tran Thiet Son. Ph.D. M.D.

## Provenance and peer review

Not commissioned, externally peer-reviewed.

## Declaration of competing interest

Authors do not report any conflict of interest.
